# Postmortem CT angiography in infants and young children (pedPMCTA): Technical feasibility and emerging forensic insights from a 30-case study

**DOI:** 10.1007/s00414-026-03750-z

**Published:** 2026-02-26

**Authors:** Jessica Vanhaebost, Bastien Trächsel, Florian T. Fischer, Silke Grabherr, Gina Maria Bruch

**Affiliations:** 1 Department of Pathology and Legal Medicine, Université Catholique de Louvain (UCLouvain) – Cliniques universitaires Saint Luc, Brussels, Belgium; 2 Pole of Morphology, Faculty of Medicine, Institute of Experimental and Clinical Research, Université Catholique de Louvain, Brussels, B-1200 Belgium; 3 University of Geneva, Rue du Général-Dufour 24, Geneva, CH - 1211 Switzerland; 4 Department of Epidemiology and Health Systems, Centre for Primary Care and Public Health (Unisanté), University of Lausanne, Lausanne, 1015 Switzerland; 5 Institute of Forensic Medicine, Ludwig-Maximilians-University Munich, Nussbaumstr. 26, D - 80336 Munich, Deutschland; 6 University Centre of Legal Medicine Lausanne-Geneva, Centre Hospitalier Universitaire Vaudois, Lausanne University, Geneva University Hospital, University of Geneva, Geneva, Switzerland; 7 Institute of Forensic Medicine Graz, Neue Stiftingtalstraße 6, Graz, 8010 Austria

**Keywords:** Postmortem CT angiography, Forensic imaging, Pediatric autopsy, Vascular imaging, Bridging veins, Infant death, PedPMCTA, PMCTA

## Abstract

**Background & objectives:**

Postmortem CT angiography (PMCTA) is increasingly used in forensic imaging but remains underexplored in pediatric populations. This study assesses the feasibility of pediatric PMCTA (pedPMCTA) using a weight-adapted manual protocol with a lipophilic contrast agent and explores its forensic application, particularly for investigating abusive head trauma.

**Materials & methods:**

Thirty pediatric cases (0–3.6 years; 2.2–25.4 kg) underwent PMCTA prior to autopsy. A biphasic protocol (arterial and venous) was applied using Angiofil™ diluted in rapeseed oil or paraffinum perliquidum. Vascular access included femoral, intraosseous, umbilical and peripheral routes. Vascular filling was assessed using a semiquantitative score for arteries (max 34) and veins (max 24). Diagnostically sufficient opacification was defined as > 17/34 for arteries and > 12/24 for veins. Access types, scores and technical success were analyzed.

**Results:**

PMCTA was technically feasible in all cases. Diagnostically sufficient arterial opacification was achieved in 23/30 cases (76.7%), and venous opacification in 24/30 (80.0%). Femoral venous access yielded the highest venous scores. In some cases, arterial opacification occurred despite venous-only injection, suggesting passive filling via arteriovenous shunts. Opacification scores showed no significant correlation with age or postmortem interval and only weak correlation with weight despite the weight-adapted protocol.

**Conclusion:**

PedPMCTA with a lipophilic contrast agent is feasible and diagnostically informative, including in neonates. It may complement forensic autopsy, especially in cases involving vascular lesions or suspected abusive injuries. These findings highlight the forensic value of pedPMCTA in determining causes of death in infants and young children.

**Supplementary Information:**

The online version contains supplementary material available at 10.1007/s00414-026-03750-z.

## Introduction

Sudden and unexplained deaths in infants and young children are among the most challenging and emotionally charged cases in forensic medicine, often raising complex diagnostic, legal and ethical questions. Autopsy remains the cornerstone of pediatric death investigations, particularly in cases involving trauma, sudden unexpected death or congenital anomalies. While postmortem imaging, especially computed tomography (PMCT), is increasingly integrated into forensic workflows, its application in children should be regarded as complementary rather than a substitute for conventional forensic autopsy [1, 2]. One major challenge during pediatric autopsy is the systematic evaluation of the vascular system, as vascular dissection is technically demanding due to the small size and anatomical variability of pediatric vessels, which may hinder the identification of subtle vascular injuries. Postmortem CT angiography (PMCTA) is an advanced imaging technique in which contrast agent is injected into the vascular system of a deceased individual, enabling high-resolution CT visualization of arteries and veins to detect hemorrhage, vascular injury or circulatory pathology that cannot or hardly be visualized by preparation at autopsy. PMCTA has proven valuable in adult forensic cases, offering detailed visualization of vascular anatomy and hemorrhagic lesions [3]. However, its use in pediatric cases remains sporadic and no standardized pediatric PMCTA (pedPMCTA) protocols currently exist. One reason for this is that pediatric autopsies pose specific technical challenges: vascular dissection is difficult as in fetuses and infants’ vascular calibres are minuscule compared with adults, requiring innovative catheterization methods and highly specialized contrast formulations to achieve adequate perfusion while limiting extravasation and artifacts. A recent study [4] demonstrated that virtual autopsy using PMCTA in fetuses and newborns with severe congenital heart or lung malformations provides a reliable and relatively simple method to diagnose complex cardiac and pulmonary anomalies, especially when great vessels are involved. Using umbilical vessel access, the authors reported strong concordance with prenatal diagnoses and conventional autopsy findings, suggesting that this technique may serve as a valuable adjunct in cases of induced termination of pregnancy, spontaneous delivery or neonatal death. A recent systematic review identified only six studies specifically addressing pedPMCTA in children from birth to 12 years of age [5]. Those studies primarily focused on feasibility and technical parameters, with most using femoral access, although alternative routes such as umbilical [6], transfontanellar [7] and intraosseous [8] have also been described. This lack of standardization is particularly critical in the context of suspected non-accidental head trauma (NAHT), where bridging vein rupture is a hallmark lesion often associated with subdural hemorrhage. However, because of their caliber and anatomical delicacy, bridging veins are often extremely difficult to identify macroscopically at autopsy [9].

The aim of this study was to evaluate the feasibility of pedPMCTA using a standardised, weight-adapted protocol with a lipophilic contrast agent, and to assess its potential contribution to forensic investigations in infants and young children, particularly in cases of suspected non-accidental head trauma (NAHT), by enabling the visualisation of critical vascular structures such as bridging veins and ocular vessels.

## Materials and methods

### Study population & data collection

This retrospective study was conducted in accordance with the Declaration of Helsinki (revised in 2013) for research involving human subjects, and was approved by the relevant ethics and/or forensic authorities. The internal protocol was approved by the local ethics committee (approval no. 19–438). A total of 30 pediatric cases who underwent PMCTA between late 2019 and mid-2025 were included. The age of the deceased ranged from 0 day to 3.66 years (median: 120.5 days), including two neonates who died intrapartum, with a mean weight of 8.2 kg (range: 2.2–25.4 kg) and a mean length of 68.7 cm (range: 43–107 cm). The postmortem interval, defined as the time between death and the PMCTA procedure, ranged from 5 to 96 h (median: 38 h). All cases were referred to the Institute of Forensic Medicine Munich, Germany and underwent PMCTA prior to prosecutor-ordered forensic autopsy. All prosecutors agreed to the complementary procedure. Inclusion criteria consisted of deceased children undergoing medicolegal investigations by PMCT and autopsy, with an intact vascular system and adequate body preservation. Collected data included demographic information (age, sex, weight and length), postmortem interval and technical details of the PMCTA procedure. Causes of death were determined by a full forensic autopsy supported and complementary examinations when indicated (histology, toxicology, microbiology…). The cause of death included infectious diseases, congenital cardiac anomalies, trauma, perinatal complications and suspected sudden infant death syndrome but excluded confirmed NAHT cases. Injection routes were mainly femoral, but also included umbilical, intraosseous and other peripheral venous access. A complete table of demographic and technical variables is provided in Table 1.

**Table 1:** Demographic and technical characteristics of the study population (*n* = 30). Variables include age, sex, weight, postmortem interval (PMI), type of vascular access, injected volumes and contrast mixture (IV= intravenous, G= Gauge, F= French, io = intraosseous, CVL= central venous line, h= hours, ml= millilitre, CM =contrast medium)

Case number	Age (days)	sex	PMI to angio (h)	Length (cm)	Weight (kg)	First Arterial injection (ml)	Second Arterial injection (ml)	Total arterial injection (ml)	Arterial access	Arterial material	First Venous injection (ml)	Second Venous injection (ml)	Total venous injection (ml)	Venous access route	venous Material	CM Mixture	Total Arterial Score	Total Venous Score
1	75,00	f	30	60	6,0	0	0	0	0	0	90	0	90	femoral	CVL (central vein)	4% Angiofil, Rapseed oil	4	19
2	459,00	f	48	80	9,2	80	0	80	femoral	IV Catheter 18G	180	40	220	io	io 20G	4% Angiofil, Rapseed oil	11	17
3	0,00	m	10	51	3,4	0	0	0	0	0	60	80	140	umbilical	IV Catheter 20G	4% Angiofil, Rapseed oil	11	14
4	64,00	m	5	63	5,4	0	0	0	0	0	110	0	110	io	io 20G	4% Angiofil, Rapseed oil	0	3
5	232,00	m	24	72	8,8	80	0	80	femoral	IV Catheter 20G	160	0	160	femoral	IV Catheter 18G	4% Angiofil, Rapseed oil	34	13
6	1,00	m	38	54	3,7	35	0	35	femoral	IV Catheter 20G	60	0	60	femoral	IV Catheter 20G	4% Angiofil, Rapseed oil	34	22
7	758,00	f	70	92	13,0	130	20	150	femoral	IV Catheter 18G	50	0	50	femoral	IV Catheter 18G	4% Angiofil, Rapseed oil	33	10
8	31,00	f	22	52	4,0	15	0	15	peripheric - other	IV Catheter 24G	40	0	40	peripheric - other	IV Catheter 24G	4% Angiofil, Rapseed oil	32	21
9	358,00	f	90	78	12,0	60	60	120	femoral	IV Catheter 20G	240	0	240	femoral	IV Catheter 18G	4% Angiofil, Rapseed oil	24	16
10	937,00	m	24	88	13,0	130	0	130	femoral	IV Catheter 18G	15	0	15	CVL	CVL	4% Angiofil, Rapseed oil	33	0
11	1059,00	f	24	94	10,4	100	0	100	femoral	IV Catheter 18G	200	0	200	io	io 20G	4% Angiofil, Paraffinum perliquidum	32	16
12	957,00	m	96	101	20,0	200	0	200	femoral	IV Catheter 18G	400	0	400	femoral	CVL	4% Angiofil, Paraffinum perliquidum	32	18
13	1336,00	m	70	91	12	0	0	0	0	0	120	200	320	CVL	CVL	4% Angiofil, Paraffinum perliquidum	17	14
14	13,00	m	29	49	3,5	35	0	35	femoral	IV Catheter 20G	70	0	70	peripheric - other	IV Catheter 20G	4% Angiofil, Paraffinum perliquidum	33	22
15	140,00	f	38	57	7,5	0	0	0	0	0	150	0	150	peripheric - other	IV Catheter 20G	4% Angiofil, Paraffinum perliquidum	11	21
16	853,00	m	83	95	18	180	0	180	femoral	Port 4 F	360	0	360	femoral	IV Catheter 18G	4% Angiofil, Paraffinum perliquidum	31	15
17	94,00	f	52	69	6,2	62	0	62	femoral	Port 4 F	124	0	124	femoral	IV Catheter 18G	4% Angiofil, Paraffinum perliquidum	31	13
18	101,00	m	48	48,5	2,6	0	0	0	0	0	50	50	100	io	io 20G	4% Angiofil, Paraffinum perliquidum	17	21
19	250,00	m	21	66	8	80	0	80	femoral	Port 5 F	0	0	0	0	0	4% Angiofil, Paraffinum perliquidum	31	0
20	53,00	f	26	57	4,1	80	0	80	femoral	IV Catheter 18G	0	0	0	0	0	4% Angiofil, Paraffinum perliquidum	30	0
21	54,00	m	53	58	4,9	50	0	50	femoral	Port 6 F	100	0	100	femoral	Port 8 F	4% Angiofil, Paraffinum perliquidum	29	16
22	434,00	m	27	65	6	60	0	60	femoral	Port 9 F	120	0	120	io	io 20G	4% Angiofil, Paraffinum perliquidum	32	17
23	163,00	f	28	61	6,2	65	0	65	femoral	IV Catheter 14G	125	0	125	femoral	IV Catheter 18G	4% Angiofil, Paraffinum perliquidum	31	20
24	43,00	f	66	53	3,6	35	35	70	femoral	IV Catheter 20G	0	0	0	0	0	4% Angiofil, Paraffinum perliquidum	32	0
25	547,00	f	86	81	10,8	110	0	110	femoral	IV Catheter 14G	220	0	220	femoral	IV Catheter 14G	4% Angiofil, Paraffinum perliquidum	32	20
26	1186,00	m	55	107	25,4	100	0	100	femoral	IV Catheter 14G	350	0	350	femoral	Port 6 F	4% Angiofil, Paraffinum perliquidum	32	13
27	88,00	m	31	64	6,2	60	0	60	peripheric - other	IV Catheter 24G	120	0	120	femoral	IV Catheter 18G	4% Angiofil, Paraffinum perliquidum	31	23
28	13,00	m	30	55	3,1	30	0	30	femoral	IV Catheter 20G	60	0	60	femoral	IV Catheter 18G	4% Angiofil, Paraffinum perliquidum	31	14
29	5,00	m	48	57	7	60	0	60	femoral	IV Catheter 20G	20	100	120	io	io 20G	4% Angiofil, Paraffinum perliquidum	33	17
30	0,00	f	48	43	2,2	0	0	0	0	0	20	40	60	umbilical	IV catheter 20G	4% Angiofil, Paraffinum perliquidum	31	22

### Preparation of the vascular access – site of injection

Before the radiological examination, an external inspection of the body was performed, followed by placement of the vascular cannulas. If intravascular catheters e.g. from emergency care were already in place at the time of death and remained in situ upon arrival at the forensic institute, they were used for contrast agent injection (seven cases). Otherwise, peripheral vascular access was established, most commonly via femoral artery and vein dissection. Femoral access was preferred whenever feasible, as it allows direct vascular cannulation with minimal disruption to central anatomical structures. If no vascular access was in place, the accesses were inserted in the femoral vessels or, for venous vascular access, intraosseously according to the published scheme [8]. The peripheral arterial and venous access was established using age-appropriate intravenous catheters (14G to 24G; Vasofix^®^ Braunüle, B. Braun Melsungen AG, Melsungen, Germany). In addition, vascular introducer sheaths (4 F to 9 F) were used depending on the subject’s anatomy, for both arterial and venous access (Terumo^®^, Leuven, Belgium). In six cases where peripheral venous access was not feasible due to vessel fragility or anatomical constraints, intraosseous access was performed using a 20 G, 15 mm intraosseous needle (Arrow^®^ EZ-IO^®^, Teleflex Inc., Morrisville, NC, USA). In some cases, due to the small caliber and previous clinical puncture attempts of the femoral vein, intraosseous access was preferred for venous contrast administration when dissection was not feasible. In two cases, the umbilical vein was still accessible and was used for venous phase injection placing intravenous catheters, as the children died during birth [Table 1].

### Imaging protocol

PMCTA was performed using a GE Revolution CT 660 system in 27 cases and a Canon Aquilion Prime SP (128-slice) system in 3 cases. A native CT (120 kV, 50 mA, Tilt 0,0, Slice 1,5 mm until 2023; then 0,675 mm) preceded the angiography (120 kV, 100 mA, 50 eff.mA, Tilt 0,0, Slice 0,675 mm). Subjects were scanned in the supine position. The imaging protocol included the following phases: (a) a non-contrast CT acquisition, (b) an arterial phase, and (c) a venous phase. In some cases, only one contrast-enhanced phase (arterial or venous) was successfully performed. Scan parameters included slice thickness 0.625 mm, tube voltage 120 kVp and reconstruction algorithms such as maximum intensity projection (MIP) and multiplanar reconstructions (MPR).

### Injection protocol

Once unenhanced multidetector CT (MDCT) images were acquired, the needed contrast agent mixture was created. A lipophilic contrast medium, specifically designed to minimize postmortem extravasation (Angiofil™, Fumedica, Switzerland), was combined with either rapeseed oil (10/30 cases – viscosity at 20 °C 74 mm²/s) or paraffinum perliquidum (20/30 cases – viscosity at 20 °C 36 mm²/s) at a 4% dilution ratio. The contrast mixture was prepared in advance in a container and drawn up as needed. The volume of contrast agent was calculated based on body weight: 10 mL/kg for arterial injection and 20 mL/kg for venous injection, according to the institutional protocol. After injection of the arterial volume and computed tomography imaging, the venous volume was injected. If an arterial injection was not possible, only a venous injection of contrast agent was performed. Injections were performed manually using 10 mL syringes.

### Image Analysis

CT images were reviewed and post-processed using OsiriX MD (version 14.0.1; Pixmeo SARL, Bernex, Switzerland). Image interpretation was performed in consensus by two board-certified forensic pathologists with expertise in forensic radiology [JV, GB]. Vascular opacification was assessed for each case and for each contrast-enhanced phase (arterial and venous), using a semi-quantitative scale as follows: 0: no opacification − 1: partial opacification (≥ appx. 50%) − 2: complete opacification (> appx. 80%). A score of “1” was also assigned when only one of two paired vessels (arteries or veins) showed complete opacification.

A total vascular opacification score was calculated for each subject across predefined anatomical regions, including the cerebral arteries (anterior, middle, and posterior cerebral arteries), ophthalmic and central retinal vessels, the basilar trunk, carotid (external, internal, and common), vertebral and jugular veins, the aortic arch and descending aorta, coronary ostia, pulmonary arteries, superior longitudinal sinus and bridging veins, abdominal vessels (superior mesenteric, renal arteries and veins, hepatic veins, inferior vena cava), and peripheral vessels (common iliac, brachial and femoral arteries and veins). Particular attention was paid to venous structures relevant to forensic assessment (e.g., bridging veins, orbital and ocular vascularization). For bridging veins, a partial score was given when only the frontal bridging veins were visualized. The central retinal vessels were considered as a single unit as it was not possible to differentiate between arterial and venous vessels postmortem by imaging. The presence of an arteriovenous communication was assessed in all cases.

### Statistical analysis

The measures of interest were reported as mean values or median values, depending on whether when the data distributions were deviated from a normal distribution according to a quantile-quantile plots. For each deceased who received contrast agent, scores were computed that summed the semi-quantitative vascular opacification of the different vessels separately for arteries and veins. Linear models were used to assess the association between the weight, the mixture, the injection site, the time before the angiography and the scores. The parameters, 95% confidence intervals and associated p-values were estimated. The normality of the residuals was assessed using quantile-quantile plots. Language refinement was supported through AI-assisted copy editing using ChatGPT (OpenAI), limited strictly to improvements in grammar and style.

## Results

### Technical feasibility

All 30 subjects received at least one injection, either during the arterial or venous phase. The main technical limitation was extravasation at the injection site and problems during preparation due to the small vessel diameter and previous clinical puncture attempts.

**Fig. 1:**
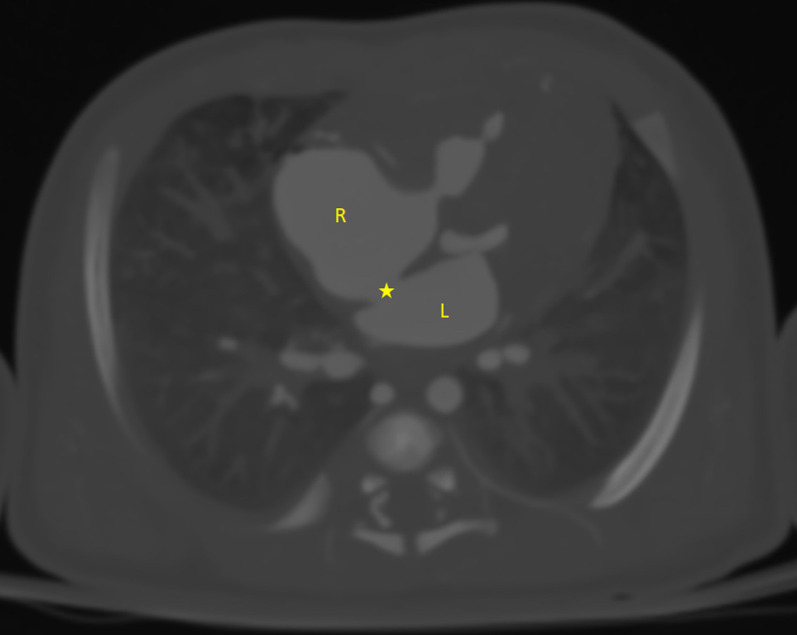
pedPMCTA in a 2-week-old male infant (weight: 3.1 kg), following sequential femoral arterial and then venous injections of lipophilic contrast agent. Axial contrast-enhanced CT image showing contrast passage from the right atrium (R) to the left atrium (L) through a patent foramen ovale (yellow star), suggesting intracardiac shunting. In this case, arterial enhancement was improved following venous injection, likely due to the presence of this communication. This observation highlights the impact of physiological postnatal shunts on vascular distribution in pedPMCTA

Arteriovenous (AV) shunts were clearly observed in 8/30 cases. In *n* = 2 cases a big open foramen ovale was observed [Figure 1], but an arterial and venous injection could be carried out as planned. In *n* = 6 of these eight cases, due to the AV-Shunt, only a venous injection was successfully carried out to fill the arterial vessel retrogradely. In these eight cases, where there was no arterial injection, the venous injection was the first and only one. In six out of these eight cases, an arterial opacification followed due to the AV shut which resulted in a simultaneous filling of the entire vessel system.

In *n* = 4, a single venous injection was sufficient to achieve satisfactory vascular opacification, also in the arterial system. In *n* = 1 the only venous injection with an AV shut produced a poor arterial filling, but in one other case (*n* = 1), the retrograde filling led to a nearly completely filled arterious and venous system. In *n* = 20 cases a separate arterial and venous injection was possible. A total of *n* = 26 cases had a fully assessable vascular system. *N* = 1 had no AV shunt and no arterial injection, so in this case, the venous phase lead only to an assessable venous vessel system. In *n* = 3 cases an arterial injection took successfully place but a venous injection of contrast agent mixture failed due to technical problems or extravasations.

Arterial access was achieved predominantly via the femoral artery (*n* = 21), followed by other peripheral arterial access (*n* = 2). Venous access was obtained through femoral (*n* = 14), intraosseous (*n* = 6), umbilical (*n* = 2), other peripheral venous access (*n* = 3) or central venous lines (*n* = 2). The mean volume of contrast injected was 84,9 mL for the arterial phase (range: 15–200 mL), and 152,7 mL for the venous phase (range: 15–400 mL), yielding a total mean injection volume of 206.4 mL (range: 55–600 mL).

### Vascular opacification

**Fig. 2:**
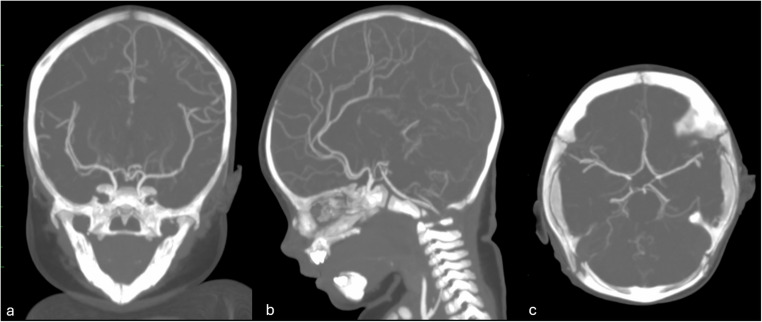
Arterial-phase pedPMCTA in a 7-week-old girl, performed via femoral arterial access. Multiplanar MIP reconstructions of the head demonstrate diagnostically sufficient opacification of the internal carotid arteries, circle of Willis, basilar artery, posterior cerebral arteries, middle cerebral arteries, and anterior cerebral arteries. (**a**) Coronal view; (**b**) Sagittal view; (**c**) Axial view. These images illustrate the feasibility of detailed cranial vascular assessment in neonates and young infants using the pedPMCTA protocol

**Fig. 3:**
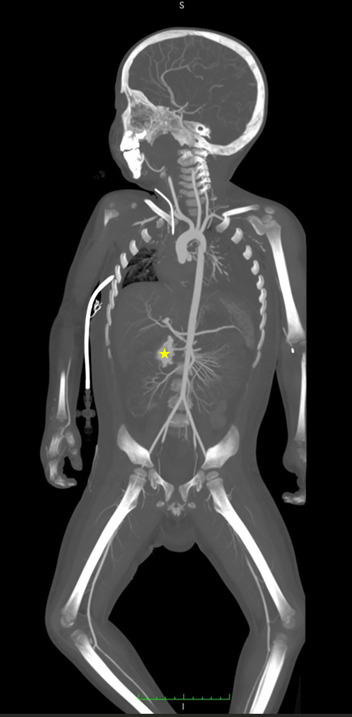
Arterial-phase pedPMCTA in a 2.5-year-old boy, performed by manual injection of 130 mL of contrast medium via femoral arterial access. Coronal MIP reconstruction shows excellent contrast opacification of the entire arterial system, including the aortic arch and its cervical branches, thoracoabdominal aorta, limb arteries, and cerebral vessels. A contrast leakage is visible (star) in the anatomical region of the right adrenal gland. This case illustrates the feasibility of comprehensive vascular imaging in older pediatric subjects using the pedPMCTA protocol

The mean arterial opacification score was 27,4 out of 34, and the mean venous score was 16,8 out of 24. Details of the evaluation of the vascular opacification can be seen in Table 3. Diagnostically sufficient opacification was defined as the visualization of more than 50% of the expected vascular territory (i.e., a total score strictly greater than 17/34 for arteries and 12/24 for veins). This threshold was chosen based on the semi-quantitative scoring system, where a score of “2” reflects complete opacification and reflects diagnostically sufficient vascular filling in the pediatric postmortem setting. For instance, a score of 2 in the cerebral arteries corresponded to full delineation of the Circle of Willis [Figure 2] and its major branches. Using this definition, diagnostically sufficient arterial opacification was achieved in 23 out of 30 cases (76.7%) and diagnostically sufficient venous opacification in 24 out of 30 cases (80%). A representative whole-body arterial-phase pedPMCTA is shown in Fig. 3.

**Fig. 4:**
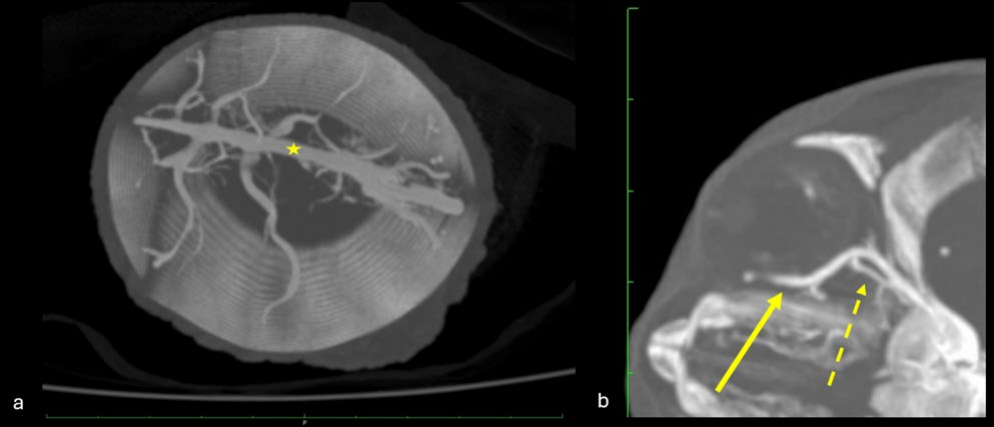
Venous-phase pedPMCTA in a neonate who died in the immediate postnatal period (weight: 2.2 kg), following umbilical venous injection of 60 mL of lipophilic contrast agent. (**a**) Axial MIP reconstruction showing the superior sagittal sinus filled with contrast (star), from which bridging veins emerge. (**b**) Axial MIP reconstruction of the orbit showing opacified ophthalmic vessel (solid arrow) and central retinal vessel arising from it (dashed arrow). This case illustrates the ability of venous-phase pedPMCTA to depict cerebral and ocular venous structures relevant in forensic

For the arteries, no significative association was found between the scores and arterial access, mixture, weight or postmortem interval. For the veins, each additional kilogram in weight was associated significatively with a decrease of 0.57 points in score (*p* = 0.01). Other variables were not significatively associated with the venous scores [Table 2]. For bridging veins [Figure 4] no significative association was found. Coronary ostia were visualized in 26/30 cases (86.6%), bridging veins and central retinal vessels in 13/30 (43.3%), while the ophthalmic vein and artery were opacified in 21/30 (70.0%) and 8/30 (26.7%) cases.

**Table 2:** Results of linear regression models assessing the association between vascular opacification scores and technical or demographic variables in (**a**) arterial phase and (**b**) venous phase. Coefficients, standard errors (SE), 95% confidence intervals (95% CI), and p values are shown

Parameter	Coefficient	SE	95% CI	*p*
(a) Arterial Phase				
(Intercept)	32.48	2.59	(27.03, 37.92)	< 0.001
Arterial access (peripheric - other) (ref: femoral)	1.09	3.82	(−6.92, 9.11)	0.778
4% Angiofil, Rapseed oil (ref: paraffinum)	−2.91	2.25	(−7.64, 1.82)	0.213
Weight (kg)	0.09	0.22	(−0.36, 0.55)	0.668
PMI (h)	−0.04	0.05	(−0.15, 0.07)	0.435
(b) Venous Phase				
(Intercept)	17.53	2.56	(12.23, 22.84)	< 0.001
Venous access (not io) (ref: io)	1.58	2.25	(−3.09, 6.25)	0.490
4% Angiofil, Rapseed oil (ref: paraffinum)	−3.82	1.96	(−7.88, 0.24)	0.064
Weight (kg)	−0.57	0.20	(−0.98, −0.15)	0.010*
PMI (h)	0.08	0.05	(−0.02, 0.18)	0.110

Femoral venous access showed the highest mean venous opacification score, followed by other peripheral and intraosseous routes. Central venous lines resulted in heterogeneous outcomes, with some complete failures. Interestingly, arterial opacification was observed in certain cases despite the absence of arterial injection. In these instances, passive arterial filling from venous circulation via arteriovenous shunts was observed.

### Artefacts and limitations

Common artefacts [10] included extravasation of contrast agent (*n* = 4), air bubbles within vascular structures (*n* = 6), and contrast sedimentation (*n* = 2). In 7 of 30 cases, only a venous injection was performed. An arterial injection was skipped due to AV-shunt, technical failure or extravasation during attempted arterial access. In six of these seven cases the arterial system was partly or fully filled with contrast agent mixture due to an AV shunt. These artefacts variably affected the interpretability of the images, particularly in smaller or peripheral vessels. In some cases, inadequate injection technique or prolonged PMI contributed to reduced vascular filling or increased background signal. Nonetheless, none of these limitations systematically prevented the evaluation of major thoracic, abdominal or cerebral vascular structures.

## Discussion

While PMCTA protocols are now well established in adults [11–13] and have shown promising feasibility in fetal applications [14–16], their adaptation to pediatric populations remains limited by anatomical and technical challenges. Those include a broad range of body sizes, tiny vascular lumen and difficulties in establishing reliable vascular access [6, 8]. The present study sought to evaluate a tailored, weight-adapted PMCTA protocol in small children and to assess its feasibility, diagnostic performance and potential forensic applications. Compared with conventional autopsy, pedPMCTA enables rapid and non-destructive visualization of vascular structures that are otherwise extremely difficult to access by dissection, such as intracranial venous pathways or small vessels. It also preserves specimen integrity, which could be important in medical autopsy cases when family consent is limited.

### Feasibility and technical considerations

This study supports the feasibility of pedPMCTA using a manual, weight-adapted injection protocol with a lipophilic contrast agent across a broad range of infants, toddlers and postmortem intervals. Our approach enabled very good intravascular filling in the vast majority of cases, despite anatomical variability and the inherent small caliber of pediatric vessels. Indeed, in neonates and infants, vascular opacification can be technically challenging due to small vessel calibers. However, diagnostically sufficient arterial filling was achieved in most cases (26 out 30 cases), including very young diseased [Fig. 2]. Angiofil™ [17], diluted in rapeseed oil or paraffinum perliquidum, proved suitable for small-body imaging. Although the type of oil mixture used in our study did not show a statistically significant association with vascular opacification [Table 2], the lower viscosity of paraffin perliquidum (36 mm²/s at 20 °C) compared with rapeseed oil (74 mm²/s at 20 °C) may indicate that paraffinum perliquidum is better suited for achieving homogeneous vascular filling.

Previous experimental studies have shown that µAngiofil™, a polymer-based contrast agent, enables a novel postmortem microangio-computed tomography (microangioCT) approach with exceptionally high spatial resolution, allowing the visualization of very small vessels. Because it can effectively perfuse vessels across a wide range of calibers, this agent holds promise for extending postmortem angiographic applications beyond the pediatric setting [18]. Diagnostically usable images could be obtained even when the arterial opacification was obtained with retrograde filling of the arterial system.

### Vascular access & injection strategies

Femoral access, although technically challenging in small children, remains the most reliable peripheral route for PMCTA. It allows direct cannulation without creating artefacts in critical autopsy areas like the thoracic inlet. Its effectiveness in adults [11, 19] gives a hint for its adaptation for pediatric use and may support future standardization efforts. In our cohort, intraosseous access was used in six cases when peripheral venous access was not feasible, offering a minimally invasive alternative [8]. Umbilical vein cannulation was successful in two neonates and should be used in perinatal cases when the umbilical cord is intact [6, 20]. Standardized access strategies adapted to age and anatomy could improve reproducibility across centers [8].

### Spontaneous arteriovenous filling and shunt observation

Arteriovenous shunts were visualized in 8/30 cases [Fig. 3]. In 4 of these, a single injection yielded satisfactory arterial and venous opacification, while 2 others demonstrated partial arterial filling from a venous injection. These findings align with prior observations in perinatal imaging [21]. This suggests that under certain conditions, single-phase PMCTA protocols may be diagnostically sufficient, reducing procedure time and complexity.

### Vascular opacification and trends

Diagnostically sufficient vascular filling was achieved in 76.7% of arteries and 80% of veins. This supports the robustness of our protocol across varying ages, body sizes, and PMIs. Notably, diagnostically sufficient opacification was possible up to 96 h PMI, extending the diagnostic window beyond the previously suggested 48-hour limit [22]. Statistical analyses revealed no significant link between opacification scores and age or PMI. The absence of significant association with weight was expected, given the weight-adapted protocol. However, a negative association was observed between the children’s weight and their venous system score. This suggests that the venous system may have a weight-dependent filling pattern, and future investigations should assess whether adjusting injected contrast volumes more strictly according to body weight could improve venous visualization. This trend suggests that a fixed mL/kg approach may not fully account for variations in body composition, particularly in edematous or overweight infants. Although this is not statistically proven in our cases, there is precedent for adjusting contrast volume in the context of edema [22]. Volume adaptation should be individualized and can also justify increasing the dose when appropriate. To address this, future protocols could differentiate arterial and venous injection volumes. Arterial volume might be based on expected weight (e.g., 50th percentile using height-based charts), while venous volume could reflect actual body weight. The greater compliance of the venous system may require more contrast in cases of fluid overload, whereas arterial tone remains relatively constant. Tailoring the protocol to such variables could enhance image quality and diagnostic reliability.

### Assessment of forensically relevant structures

Radiologic and histopathologic evidence has emphasized the crucial role of venous injury, including sinus thrombosis, bridging vein rupture and retinal hemorrhages in the pathophysiology of NAHT [23]. To explore the potential of pedPMCTA in evaluating venous structures non-invasively, we assessed the superior sagittal sinus, the bridging veins and the ocular vascularization to understand if PMCTA could contribute to the non-invasive evaluation of venous structures in pediatric decedents.

#### Cerebral venous structures

We first assessed the *superior sagittal sinus*, as some authors have suggested that non-traumatic dural sinus thrombosis might mimic or coexist with NAHT-related findings [23, 24]. Among the 30 analysed cases, 15 allowed the visualization of the superior longitudinal sinus. However, it has to be noted that no deceased child older than 1,5 years or more than 12 kg body weight (each *n* = 7) had a contrasted sagittal sinus or contrasted bridging veins. This indicates that the protocol provides too little venous vessel filling with this age/weight. We therefore hypothesise that the injected volume in these cases needs to be increased, as the visualisation of these structures is important. Although non-traumatic intracranial venous thrombosis has been proposed as a differential diagnosis in NAHT, it remains rare and is typically associated with underlying risk factors such as dehydration, infection or malignancy. Nonetheless, being able to assess venous thrombosis may add value in the evaluation of ambiguous cases.

The *bridging veins*, especially susceptible to tearing under shearing stress [25], are of particular forensic interest. A large autopsy series has shown that these veins are fragile, variable in number and mainly located over the convexities of the frontal and parietal lobes [9]. Their anatomical delicacy makes them difficult to identify once the brain has been removed, and histologic confirmation of rupture is not always feasible. While conventional autopsy may overlook them, ante mortem imaging has demonstrated that bridging veins draining into the superior sagittal sinus can be visualized by CT angiography [26]. Based on these findings, PMCTA could offer a non-destructive alternative for assessing bridging veins in deceased infants [7]. In our study, bridging vein opacification was achieved in 13 out of 30 cases (43%), supporting the technical feasibility of their depiction, though emphasizing the need for further protocol refinement [Fig. 4a]. Identifying bridging vein rupture could significantly aid in differentiating abusive from accidental head trauma in cases of subdural hemorrhage. Notably, bridging veins were visualized in 13 of the 15 cases (87%) in which the superior longitudinal sinus was also opacified, suggesting that sinus filling may be a prerequisite for their reliable depiction. However, PMCTA’s spatial resolution may still be insufficient to detect subtile discontinuities, and findings have yet to be systematically validated by histology. Future research should correlate imaging with autopsy and investigate how technical factors, such as injection parameters or postmortem interval, impact venous detectability.

#### Ocular vessels

Retinal hemorrhages are key forensic signs but may be missed if eyes are not enucleated during autopsy. PedPMCTA offers a potential non-invasive alternative evaluation of the *ocular vascularization*. In our series, the ophthalmic veins were visualized in 70% of cases, the central retinal vessels in 43%, and the ophthalmic artery in 27% [Fig. 4b]. While PMCTA does not yet replace traditional methods, these preliminary results suggest it could contribute to postmortem evaluation of retinal hemorrhages—particularly if dedicated orbital protocols and higher-resolution acquisitions, e.g. photon-counting CT are employed. Evidence from living children indicates that magnetic resonance imaging has shown promising results in this regard [23], whereas in the postmortem setting PMCTA may provide a complementary approach, particularly when MRI or ophthalmologic expertise is not available.

### Coronary evaluation

Coronary artery anomalies, although frequently asymptomatic, are recognized causes of sudden death in infants and children [27]. In our series, coronary ostia were visualized in 26/30 cases (86.6%). The systematic identification of ostia is a promising first step toward comprehensive postmortem coronary assessment. The assessment of these important vessels will be evaluated separately. Targeted postmortem coronary angiography has already demonstrated the value of coronary imaging in neonates with congenital heart disease [28]. In the future, pedPMCTA could play a key role in postmortem cardiac imaging, especially if technical refinements allow comprehensive coronary visualization, including critical segments such as interarterial or intramural courses. These improvements will be essential to better assess the role of coronary anomalies in sudden pediatric deaths.

### Study limitations

This was a single-center feasibility study with a limited sample size and heterogeneous population, precluding detailed subgroup analysis. One limitation could be seen in the fact that the interpretation of the obtained images was performed by two forensic pathologists, without a board-certified radiologist. However, as the aim of the study was just to verify the technical approach by indicating if specific vessels real filled with contrast agent or not, we considered the knowledge of the well trained for forensic pathologists was enough to obtain this goal. Indeed, no radiological interpretation of paediatric pathology was performed in the context of this study. No comparison with autopsy findings was yet performed, so it is not possible to calculate sensitivity or specificity. Prospective studies comparing PMCTA to both macroscopic and microscopic autopsy findings are needed to validate its diagnostic accuracy. Manual contrast injection could introduce variability, and vascular leakage or incomplete filling may mimic pathology. Finally, although image quality was assessed using opacification scores, interobserver variability was not measured due to consensus reading, and scoring was based on visual interpretation. The small sample also limited the power in the scores analysis. The mixture had a similar effect on the scores of arteries and veins, albeit not significant, presumably due to the limited sample size. Further studies on larger scales would be advisable.

### Future perspectives

Despite increasing interest in pedPMCTA, no standardized protocol currently exists. The development of consensus-based guidelines is essential to ensure reproducibility and improve diagnostic consistency therefore allowing for robust intercenter comparisons. Future studies should focus on protocol optimization, including the adaptation of injection volumes to individual physiological variables, particularly in cases of edema or overweight infants, and the refinement of vascular access strategies based on age and body habitus. The routine use of dual-phase protocols also warrants reassessment, as their necessity may vary depending on case-specific goals and vascular anatomy. Multicenter collaborative efforts will be critical to validate the diagnostic performance of pedPMCTA, define reliable thresholds for adequate vascular filling, and better characterize normal and pathological postmortem vascular patterns in infants and young children.

## Conclusion

This study confirms the feasibility of pedPMCTA using a standardized, weight‑adapted protocol and demonstrates its diagnostic value in infants and young children by manually injecting through peripheric access. The vascular access should be preferably femoral if possible, injecting 10mL/kg in the arterial phase and 20mL/kg in the venous phase of a lipophilic contrast agent diluted to 4% in oil of low viscosity. When tailored to body size, pedPMCTA produces sufficient quality vascular imaging that can supplement the findings of forensic autopsy and may substantially enhance diagnostic yield, particularly for vascular lesions that are difficult to detect macroscopically. It is not intended to replace a (prosecutor-ordered) autopsy, which remains compulsory, but rather to enhance its diagnostic yield, particularly for vascular lesions that are difficult to identify macroscopically, such as bridging‑vein ruptures or subtle cardiac and retinal hemorrhages. Outside the judicial context, where a hospital (consent‑based) autopsy may be declined, pedPMCTA offers a less‑invasive alternative that can still provide valuable information for clinicians and families. Larger, multicenter studies are needed to validate these findings, refine pediatric protocols and define the precise role of pedPMCTA in both forensic and clinical post‑mortem practice.

**Table:** 

Parameter	Coefficient	SE	95% CI	*p*
(Intercept)	17.53	2.56	(12.23, 22.84)	< 0.001
Venous access (not io) (ref: io)	1.58	2.25	(−3.09, 6.25)	0.490
4% Angiofil, Rapseed oil (ref: paraffinum)	−3.82	1.96	(−7.88, 0.24)	0.064
Weight (kg)	−0.57	0.20	(−0.98, −0.15)	0.010*
PMI (h)	0.08	0.05	(−0.02, 0.18)	0.110

## Supplementary Information

Below is the link to the electronic supplementary material.

Supplementary Material 1 (DOCX 26.1 KB)

## Data Availability

The datasets generated during and/or analyzed during the current study are available from the corresponding author on reasonable request.
