# Supplementary material for: Postmortem CT angiography in infants and young children (pedPMCTA): Technical feasibility and emerging forensic insights from a 30-case study
Source: Int J Legal Med. 2026 Feb 26;140(4):2131–41. doi: 10.1007/s00414-026-03750-z (PMC13469391; doi:10.1007/s00414-026-03750-z)
Supplement: Supplementary file 1 [file 414_2026_3750_MOESM1_ESM.docx]

| Case number | Anterior cerebral artery | Middle cerebral artery | Posterior cerebral artery | ophtalmic artery | Basilar trunk | External carotid artery | Internal carotid artery | Vertebral arteries | Common carotid arteries | Aortic arch | Descending aorta | Coronary ostia | Superior mesenteric artery | Renal arteries | Common iliac arteries | Brachial arteries | Femoral arteries | TOTAL ARTERY /34 |
| --- | --- | --- | --- | --- | --- | --- | --- | --- | --- | --- | --- | --- | --- | --- | --- | --- | --- | --- |
| 1 | 0 | 0 | 0 | 0 | 0 | 0 | 0 | 0 | 0 | 2 | 1 | 0 | 0 | 0 | 0 | 1 | 0 | 4 |
| 2 | 0 | 0 | 0 | 0 | 0 | 0 | 0 | 0 | 1 | 2 | 2 | 0 | 2 | 2 | 1 | 1 | 0 | 11 |
| 3 | 0 | 0 | 0 | 0 | 0 | 0 | 0 | 0 | 0 | 2 | 2 | 1 | 2 | 0 | 2 | 0 | 2 | 11 |
| 4 | 0 | 0 | 0 | 0 | 0 | 0 | 0 | 0 | 0 | 0 | 0 | 0 | 0 | 0 | 0 | 0 | 0 | 0 |
| 5 | 2 | 2 | 2 | 2 | 2 | 2 | 2 | 2 | 2 | 2 | 2 | 2 | 2 | 2 | 2 | 2 | 2 | 34 |
| 6 | 2 | 2 | 2 | 2 | 2 | 2 | 2 | 2 | 2 | 2 | 2 | 2 | 2 | 2 | 2 | 2 | 2 | 34 |
| 7 | 2 | 2 | 2 | 1 | 2 | 2 | 2 | 2 | 2 | 2 | 2 | 2 | 2 | 2 | 2 | 2 | 2 | 33 |
| 8 | 2 | 2 | 2 | 0 | 2 | 2 | 2 | 2 | 2 | 2 | 2 | 2 | 2 | 2 | 2 | 2 | 2 | 32 |
| 9 | 1 | 1 | 1 | 0 | 0 | 1 | 1 | 2 | 1 | 2 | 2 | 2 | 2 | 2 | 2 | 2 | 2 | 24 |
| 10 | 2 | 2 | 2 | 1 | 2 | 2 | 2 | 2 | 2 | 2 | 2 | 2 | 2 | 2 | 2 | 2 | 2 | 33 |
| 11 | 2 | 2 | 2 | 0 | 2 | 2 | 2 | 2 | 2 | 2 | 2 | 2 | 2 | 2 | 2 | 2 | 2 | 32 |
| 12 | 2 | 2 | 2 | 0 | 2 | 2 | 2 | 2 | 2 | 2 | 2 | 2 | 2 | 2 | 2 | 2 | 2 | 32 |
| 13 | 0 | 0 | 0 | 0 | 0 | 0 | 0 | 1 | 1 | 2 | 2 | 1 | 2 | 2 | 2 | 2 | 2 | 17 |
| 14 | 2 | 2 | 2 | 2 | 2 | 2 | 2 | 2 | 2 | 2 | 2 | 1 | 2 | 2 | 2 | 2 | 2 | 33 |
| 15 | 0 | 0 | 0 | 0 | 0 | 0 | 0 | 0 | 0 | 2 | 2 | 2 | 2 | 2 | 1 | 0 | 0 | 11 |
| 16 | 2 | 2 | 2 | 0 | 2 | 2 | 2 | 2 | 2 | 2 | 2 | 1 | 2 | 2 | 2 | 2 | 2 | 31 |
| 17 | 2 | 2 | 2 | 0 | 2 | 2 | 2 | 2 | 2 | 2 | 2 | 1 | 2 | 2 | 2 | 2 | 2 | 31 |
| 18 | 0 | 0 | 1 | 0 | 0 | 0 | 0 | 0 | 2 | 2 | 2 | 0 | 2 | 2 | 2 | 2 | 2 | 17 |
| 19 | 2 | 2 | 2 | 0 | 2 | 2 | 2 | 2 | 2 | 2 | 2 | 1 | 2 | 2 | 2 | 2 | 2 | 31 |
| 20 | 2 | 2 | 2 | 0 | 2 | 2 | 2 | 2 | 2 | 2 | 2 | 1 | 2 | 2 | 2 | 2 | 1 | 30 |
| 21 | 2 | 2 | 2 | 0 | 2 | 2 | 2 | 2 | 2 | 2 | 2 | 1 | 2 | 2 | 1 | 2 | 1 | 29 |
| 22 | 2 | 2 | 2 | 0 | 2 | 2 | 2 | 2 | 2 | 2 | 2 | 2 | 2 | 2 | 2 | 2 | 2 | 32 |
| 23 | 2 | 2 | 2 | 0 | 2 | 2 | 2 | 2 | 2 | 2 | 2 | 1 | 2 | 2 | 2 | 2 | 2 | 31 |
| 24 | 2 | 2 | 2 | 1 | 2 | 2 | 2 | 2 | 2 | 2 | 2 | 1 | 2 | 2 | 2 | 2 | 2 | 32 |
| 25 | 2 | 2 | 2 | 0 | 2 | 2 | 2 | 2 | 2 | 2 | 2 | 2 | 2 | 2 | 2 | 2 | 2 | 32 |
| 26 | 2 | 2 | 2 | 0 | 2 | 2 | 2 | 2 | 2 | 2 | 2 | 2 | 2 | 2 | 2 | 2 | 2 | 32 |
| 27 | 2 | 2 | 2 | 0 | 2 | 2 | 2 | 2 | 2 | 2 | 2 | 1 | 2 | 2 | 2 | 2 | 2 | 31 |
| 28 | 2 | 2 | 2 | 0 | 2 | 2 | 2 | 2 | 1 | 2 | 2 | 2 | 2 | 2 | 2 | 2 | 2 | 31 |
| 29 | 2 | 2 | 2 | 1 | 2 | 2 | 2 | 2 | 2 | 2 | 2 | 2 | 2 | 2 | 2 | 2 | 2 | 33 |
| 30 | 2 | 2 | 2 | 2 | 2 | 2 | 2 | 2 | 2 | 2 | 2 | 2 | 2 | 0 | 2 | 2 | 1 | 31 |

Table 3 (a) Detailed evaluation of the vessels par case (arterial)

| Case number | Superior longitudinal sinus | Bridging veins | Jugular veins | ophtalmic vein | central retinal vessel | Heart cavity | Pulmonary arteries | Inferior vena cava | Hepatic veins | Renal veins | Brachial vein | Femoral vein | TOTAL VEIN (/24) |
| --- | --- | --- | --- | --- | --- | --- | --- | --- | --- | --- | --- | --- | --- |
| 1 | 2 | 2 | 2 | 1 | 0 | 2 | 2 | 2 | 2 | 2 | 0 | 2 | 19 |
| 2 | 2 | 1 | 2 | 1 | 0 | 2 | 2 | 2 | 2 | 2 | 0 | 1 | 17 |
| 3 | 0 | 0 | 1 | 2 | 1 | 2 | 2 | 2 | 2 | 2 | 0 | 0 | 14 |
| 4 | 0 | 0 | 0 | 0 | 0 | 1 | 0 | 0 | 1 | 1 | 0 | 0 | 3 |
| 5 | 0 | 0 | 0 | 0 | 1 | 2 | 2 | 2 | 2 | 2 | 0 | 2 | 13 |
| 6 | 2 | 1 | 2 | 2 | 1 | 2 | 2 | 2 | 2 | 2 | 2 | 2 | 22 |
| 7 | 0 | 0 | 0 | 0 | 1 | 1 | 1 | 2 | 2 | 2 | 0 | 1 | 10 |
| 8 | 2 | 2 | 2 | 2 | 1 | 2 | 2 | 2 | 2 | 2 | 1 | 1 | 21 |
| 9 | 1 | 0 | 2 | 2 | 0 | 1 | 2 | 2 | 2 | 2 | 0 | 2 | 16 |
| 10 | 0 | 0 | 0 | 0 | 0 | 0 | 0 | 0 | 0 | 0 | 0 | 0 | 0 |
| 11 | 0 | 0 | 2 | 2 | 0 | 2 | 2 | 2 | 2 | 2 | 0 | 2 | 16 |
| 12 | 0 | 0 | 1 | 2 | 1 | 2 | 2 | 2 | 2 | 2 | 2 | 2 | 18 |
| 13 | 0 | 0 | 2 | 2 | 0 | 2 | 2 | 2 | 2 | 2 | 0 | 0 | 14 |
| 14 | 2 | 2 | 2 | 2 | 2 | 2 | 2 | 2 | 2 | 2 | 1 | 1 | 22 |
| 15 | 2 | 2 | 2 | 2 | 1 | 2 | 2 | 2 | 2 | 2 | 0 | 2 | 21 |
| 16 | 0 | 0 | 2 | 2 | 0 | 1 | 2 | 2 | 2 | 2 | 0 | 2 | 15 |
| 17 | 0 | 0 | 2 | 0 | 0 | 1 | 2 | 2 | 2 | 2 | 0 | 2 | 13 |
| 18 | 2 | 2 | 2 | 2 | 1 | 2 | 2 | 2 | 2 | 2 | 0 | 2 | 21 |
| 19 | 0 | 0 | 0 | 0 | 0 | 0 | 0 | 0 | 0 | 0 | 0 | 0 | 0 |
| 20 | 0 | 0 | 0 | 0 | 0 | 0 | 0 | 0 | 0 | 0 | 0 | 0 | 0 |
| 21 | 1 | 0 | 2 | 1 | 0 | 2 | 2 | 2 | 2 | 2 | 0 | 2 | 16 |
| 22 | 2 | 1 | 1 | 2 | 0 | 2 | 2 | 2 | 2 | 2 | 0 | 1 | 17 |
| 23 | 2 | 1 | 2 | 2 | 1 | 2 | 2 | 2 | 2 | 2 | 0 | 2 | 20 |
| 24 | 0 | 0 | 0 | 0 | 0 | 0 | 0 | 0 | 0 | 0 | 0 | 0 | 0 |
| 25 | 2 | 2 | 2 | 2 | 0 | 2 | 2 | 2 | 2 | 2 | 0 | 2 | 20 |
| 26 | 0 | 0 | 1 | 0 | 0 | 2 | 2 | 2 | 2 | 2 | 0 | 2 | 13 |
| 27 | 2 | 2 | 2 | 2 | 1 | 2 | 2 | 2 | 2 | 2 | 2 | 2 | 23 |
| 28 | 0 | 0 | 1 | 2 | 0 | 2 | 1 | 2 | 2 | 2 | 0 | 2 | 14 |
| 29 | 2 | 2 | 1 | 2 | 1 | 2 | 2 | 2 | 2 | 0 | 0 | 1 | 17 |
| 30 | 2 | 2 | 2 | 2 | 2 | 2 | 2 | 2 | 2 | 2 | 2 | 0 | 22 |

Table 3 (b) Detailed evaluation of the vessels par case (venous)
